# Construction of Urban Environmental Performance Evaluation System Based on Multivariate System Theory and Comparative Analysis: A Case Study of Chengdu-Chongqing Twin Cities, China

**DOI:** 10.3390/ijerph20054515

**Published:** 2023-03-03

**Authors:** Liang Chen, Huan Huang, Dong Yao, Haonan Yang, Shuangshuang Xu, Shiyu Liu

**Affiliations:** 1 School of Finance and Accounting, Chengdu Jincheng College, Chengdu 610097, China; 2 Postdoctoral Station of Management Science and Engineering, Chengdu University of Technology, Chengdu 610059, China; 3 College of Business, Chengdu University of Technology, Chengdu 610059, China

**Keywords:** environmental performance evaluation, environmental governance, combined weighting method, multivariate system

## Abstract

Based on the related environmental data of Chengdu and Chongqing from 2011 to 2020, this paper constructs a multivariate environment performance evaluation system, combines the self-built indicator system determination criteria and rules, evaluates and compares the environmental performance of Chengdu and Chongqing, and also discusses the impact of COVID-19 on urban environmental performance. The research results show that the overall environmental performance increased from 2011 to 2020, but there are differences between different subsystems, mainly manifested in the best water environment performance, followed by air environment and solid waste; moreover, the noise environment maintains a relatively stable level. By comparing the average levels of various subsystems of the Chengdu-Chongqing dual cities from 2011 to 2020, it can be seen that Chengdu City has better environmental performance in air environment and solid waste, while Chongqing City has better environmental performance in the water environment and noise environment. In addition, this paper also found that the impact of the epidemic on urban environmental performance mainly comes from the impact on the air environment. At present, the overall environmental performance of the two places has shown a trend of environmentally coordinated development. In the future, Chengdu and Chongqing should further optimize and improve their relatively weak environmental subsystems, deepen the joint action mechanism between the two places, and build a green and high-quality development economic circle for the Chengdu-Chongqing twin cities.

## 1. Introduction

Environmental conditions have gradually become one of the focuses of global attention since the end of the twentieth century [1]. The United Nations released the Millennium Development Goals (MDGs) and Sustainable Development Goals (SDGs) in the twenty-first century, in 2000 and 2015, respectively [2,3]. Among these objectives, environmental regulation has been mentioned numerous times, demonstrating the importance of the environment in the future development of humanity. With the intensification of the degree of harm to the human living environment caused by climate change [4], all sectors of society’s attention to global environmental regulation have been heightened [5], and humanity must re-examine its concept of environmental regulation and make greater efforts for it. As a result, it is critical to improve the environment, protect the human living environment, and promote long-term development.

According to statistics, environmental pollution in China causes an estimated 10% of the country’s GDP to be lost annually [6], and not only does it reduce overall economic growth, but it also resists the quality of that growth. Since 2012, the Chinese government has made ecological civilization construction a priority for achieving sustainable development [7], which is an essential national development strategy. A significant amount of capital is invested every year in the construction of China’s ecological civilization for a variety of environmental quality improvement projects, such as ecological restoration and pollution control. At the same time, the government has also made corresponding adjustments to the industrial side. By directing capital flows to green industries and other means [8], it encourages technological innovation in green and more sustainable industries [9] and supports the vigorous development of such enterprises, which in turn gradually transforms the current market allocation and efficiency of production factors, thus driving industrial renewal and promoting the upgrading of industrial structures [10].

Cities are not only the primary drivers of regional economic growth but also the primary source of pollution [11,12]. It is possible to advance the environmental sustainability of the entire region and even the world by promoting improvements to the environmental conditions of cities. Therefore, cities play an important role in ecological and environmental management. The Chengdu-Chongqing urban agglomeration, as the central urban agglomeration in western China, has been approved as one of the state’s first three inter-regional urban agglomerations to be built [13]. It has always been a state priority and an important source of support for China’s economic growth, taking the lead in promoting rapid economic growth in western regions. Developing the Chengdu-Chongqing economic circle vigorously drives the development of cities in the Chengdu-Chongqing region, which eventually spreads throughout western China [14]. Chengdu and Chongqing, as the core cities of the Chengdu-Chongqing region, not only promote their development by absorbing production factors from surrounding cities but also drive the economic development of surrounding areas through spillover effects [15], thus relieving the burden of resources in the surrounding areas and contributing to the improvement of the ecological environment and ecological civilization construction level in the surrounding areas [16,17]. In recent years, with the closer cooperation between Chengdu and Chongqing, the local government has been paying more attention to the synergistic development of Chengdu and Chongqing. Strengthening the level of ecological civilization in Chengdu and Chongqing can help provide better development opportunities for residents and make Chengdu and Chongqing more competitive. Therefore, based on the above background, this study conducts a comprehensive assessment of the environmental conditions of Chengdu and Chongqing from the perspective of the urban environment, explores whether the developmental trends of the environmental conditions of the two regions are similar, analyzes the internal linkages between them, and thus provides suggestions for the relevant authorities on the construction of ecological civilization in the Chengdu-Chongqing region, as well as a reference for exploring the sustainable development of large global urban agglomerations.

In order to quantify the regional environmental conditions, established studies usually choose a single indicator or construct an evaluation indicator system for comprehensive measurement and analysis [18]. Among them, single indices are usually used in some empirical studies to represent the environmental conditions of the study area through a specific indicator as a variable in the measurement model, e.g., the annual average concentration of PM2.5 [19], sulfur dioxide emissions [20], etc. The comprehensive indicator systems can examine the regional environment from multiple dimensions and are usually used to evaluate and analyze the overall environmental conditions [21]. Before conducting a comprehensive evaluation, it is necessary to standardize and assign weights to each indicator. Currently, the methods of weight assignment can be divided into three types: subjective weighting, objective weighting, and a combination of subjective and objective weighting. Currently, the more commonly used subjective assignment methods are: the analytic hierarchy process (AHP) method [22], the decision-making trial and evaluation laboratory (DEMATEL) method [23], etc.; the commonly used objective assignment methods are: the entropy method [24], the CRITIC method [25]; and the combination of subjective and objective assignment methods. Because this method can minimize the loss of information and make its weight value closer to reality, more and more comprehensive evaluation-related studies are choosing the combination weighting method to assign the weights of indicators. For example, the multiplicative combination method is used to combine the subjective and objective weights [26], or the assignment process is transformed into a single-objective optimization problem with the objective function of minimizing the deviation between the combined weights and the subjective and objective weights, which is solved by using game theory [27].

**Figure 1:**
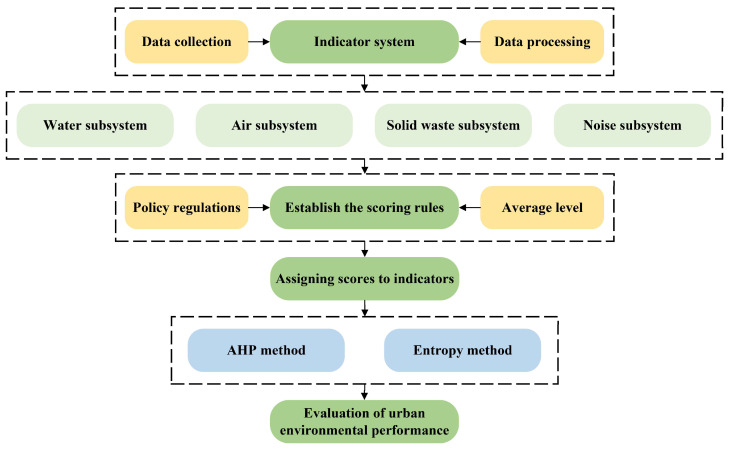
Diagram of the research path.

To summarize, the majority of existing studies on the evaluation of urban environmental performance have built indicator systems and used comprehensive evaluation models to conduct comprehensive assessments of urban environmental performance. However, there is relatively little literature on assessing environmental performance by referring to policy documents and establishing scoring criteria, for example, completing the assessment of environmental performance in the twin cities of Chengdu and Chongqing. Therefore, this study takes the twin cities of Chengdu and Chongqing as the research object and constructs a new multifaceted urban environmental performance evaluation system with four dimensions: air environment, water environment, solid waste, and noise environment, using a combination of subjective and objective weighting methods to build, referring to the existing national standards of urban requirements at all levels, industry norms, planning documents, and other policies and regulations, establishing the criteria and rules for determining the indicators of urban environmental performance evaluation system, evaluating and comparing the urban environmental performance of Chengdu and Chongqing, and thoroughly studying the trends of performance changes of different environmental subsystems and influencing factors in the past ten years (Figure 1). Furthermore, the main goal of this research is to provide a new set of scientific and reasonable indicator systems for future studies on urban environmental performance assessment, as well as a reference for future studies by other scholars in terms of evaluation methods.

## 2. Materials

### 2.1. Study Area

**Figure 2:**
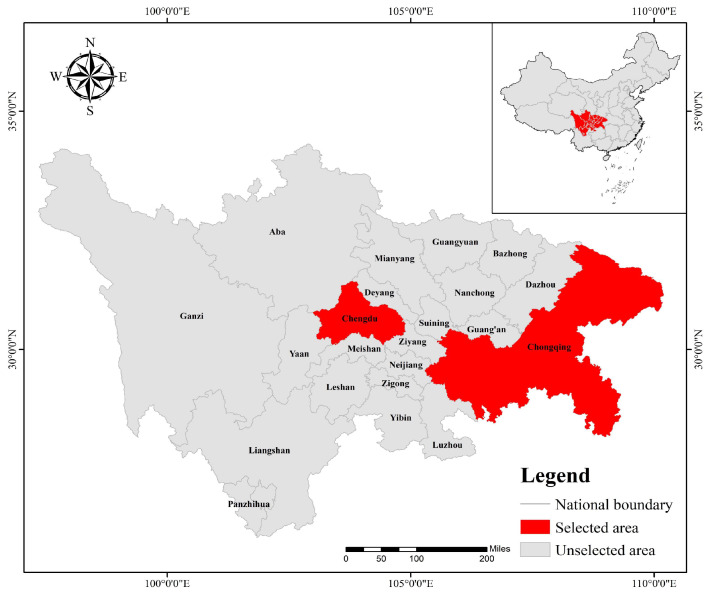
Study area.

The Chengdu-Chongqing region is located in western China and is an important support for China’s inland economic development, while the Chengdu-Chongqing twin cities, as the core cities of the Chengdu-Chongqing region, play a significant role in promoting the economic and social development of the entire Chengdu-Chongqing twin-cities economic circle. Chengdu is located in Sichuan Province, bordering the cities of Deyang, Ziyang, Meishan, and Ya’an to the east and Aba Tibetan and Qiang Autonomous Prefectures to the west. The longitude and latitude of its location are between 102°54′–104°53′ east and 30°05′–31°26′ north. Chongqing borders Sichuan Province and is located in the eastern part of Sichuan Province, in the upper part of the Yangtze River Economic Belt, which borders Shaanxi Province to the north, Hubei Province and Hunan Province to the east, and Guizhou Province to the south. The longitude and latitude in which it is located are between 106°59′–108°30′ east and 28°07′–29°39′ north (Figure 2).

### 2.2. Data Resources

In this study, the data related to the urban environment from 2011–2020 in Chengdu and Chongqing were selected for the base period of 2011, respectively. The original data of all indicators in this study were obtained from the China Statistical Yearbook (2012–2021), China Environmental Statistical Yearbook (2012–2021), China Urban and Rural Construction Statistical Yearbook (2012–2021), Sichuan Provincial Statistical Yearbook (2012–2021), Chongqing Municipal Statistical Yearbook (2012–2021), Chengdu City Ecological Environment Quality Bulletin (2011–2020), and Chongqing Ecological Environment Statistical Annual Report (2011–2020).

### 2.3. Construction of Environmental Performance Evaluation Indicator System

In constructing the environmental indicator system, most studies typically follow the theoretical framework of the PSR model [16,28]. Here, P stands for the pressure on the environment caused by human activities, S indicates the current environmental status, and R represents the response measures taken by humans to protect the environment [29]. Other scholars have created indicator systems with three dimensions: solid, liquid, and gas, with noise sometimes included in the scope of their studies [30]. For this study, we chose the second framework, which is to develop an urban environmental performance evaluation indicator system based on four dimensions: water, atmosphere, solid waste, and noise.

In terms of the air environment, PM2.5, PM10, sulfur dioxide concentration, and nitrogen dioxide concentration are important components of the air quality indicator (AQI) [31]. However, since PM2.5 was only made a Chinese air routine monitoring indicator in 2013, it was removed from the indicator system in this study and replaced with PM10 to reflect the concentration of particulate matter in the air. At the same time, the number of days with air quality reaching level two or above, the proportion of days with excellent air quality throughout the year, and the capacity of waste gas treatment equipment were used to measure the cities’ response capability in air environmental management. In addition, this study also takes into account the global warming caused by climate change [32], and the annual average temperature is also included in the evaluation indicator system of the air environment subsystem.

In terms of the water environment, this research creates an indicator system based on water supply and drainage. So as to take into account the influence of urban population and other factors, relative indicators are preferred over absolute indicators as much as possible. At the same time, a distinction is made between domestic and industrial water use to ensure that the results are fair. Water supply is represented by the urban water reuse rate, urban water saving rate, industrial water reuse rate, industrial water saving rate, per capita comprehensive water consumption, and unit GDP water consumption. For drainage, two independently constructed indicators, namely unit urban sewage discharge intensity (total urban sewage discharge/GDP) and unit industrial sewage discharge intensity (total industrial sewage discharge/GDP), are used for evaluation.

For solid waste, this study distinguished the categories of solid waste according to their sources and classified them into industrial solid waste and domestic solid waste. For industrial solid waste, industrial solid waste generation, industrial solid waste comprehensive utilization, and the industrial solid waste comprehensive utilization rate were selected; for domestic solid waste, total domestic waste removal, domestic waste harmless treatment rate, domestic waste harmless treatment rate, domestic waste removal per capita and domestic waste harmless treatment capacity was selected. In addition, for the noise aspect, from the current public data, the monitoring of noise is measured by the two indicators of urban road traffic noise and urban environmental noise, so it is chosen as the evaluation indicator for measuring the noise subsystem in this paper.

In summary, this study will take the air environment subsystem, water environment subsystem, solid waste subsystem, and noise environment subsystem environmental performance indicators as the primary indicator and subdivide the corresponding primary indicator into 24 secondary indicators concerning relevant domestic and international policies to establish a comprehensive evaluation system of the multivariate environmental performance of the Chengdu and Chongqing twin cities, and the specific indicators are detailed in Table 1.

## 3. Methodology

### 3.1. AHP Method

The analytic hierarchy process (AHP) method is used in this paper to assign weights to the urban environmental performance evaluation indicator system, which has the advantages of simple operation, easy understanding of its principles, and enhanced scientific rationality of the assignment results through the subjective judgment of experts and scholars. As a result, this method has broad applicability in the solution process for problems with multiple criteria and objectives, and it is widely used in the comparative study of multiple schemes [33,34,35,36], primarily building an expert subjective evaluation matrix for hierarchical scoring comparison and then accounting for the relevant indicators to determine weight assignment.

(1) Constructing the judgment matrix

A team of experts from universities, design institutes, research institutes, etc., who have a better understanding of urban environmental assessment, conduct independent surveys and comparative studies based on information about the environmental characteristics of the Chengdu-Chongqing region to complete the relevant questions of the questionnaire for scoring while constructing a judgment matrix to determine the scale, as shown in Equation (1).

(1) $$A=\left[\begin{array}{cccc} a_{11} & a_{12} & \cdots & a_{1m}\\ a_{21} & a_{22} & \cdots & a_{2m}\\ \vdots & \vdots & \ddots & \vdots \\ a_{n1} & a_{n2} & \cdots & a_{nm} \end{array}\right]$$

The geometric mean method is used in this paper to synthesize the 10 judgment matrices collected from experts, and finally, a judgment matrix is formed, in which $a_{nm}$ indicates the importance of the n-th level indicator relative to the m-th level indicator, based on the importance rating between the above indicators, and thus the specific contents obtained are organized as follows (Table 2).

(2) Calculation of subjective weight

In order to improve the accuracy of feature vector assignment, this paper chooses the sum-product method to complete the corresponding calculation. Firstly, each column of the judgment matrix A is processed by Equation (2) to determine the new matrix $B={\left(b_{ij}\right)}_{n\times m}$.

(2) $$b_{ij}=\frac{b_{ij}}{\sum_{i=1}^{n}b_{ij}}$$

The matrix B processed by Equation (2) is summed up row by row by Equation (3) to obtain the weights $w_{i}$ without normalization. Then, the weights $w_{i}$ are normalized according to Equation (4) to obtain the final subjective weight results $W_{i}$.

(3) $$w_{j}=\sum_{j=1}^{m}b_{ij}$$

(4) $$W_{j}=\frac{w_{j}}{\sum_{j=1}^{m}w_{j}}$$

(3) Consistency testing

In addition, to verify whether the expert scoring logic guarantees consistency and thus ensures the final assignment results are reasonable and valid, this study conducted a consistency test on the assignment results [37]. Meanwhile, to improve the accuracy of the feature vector assignment, the sum-product method is chosen to complete the corresponding calculation in this paper.

The maximum eigenvalue $\lambda_{max}$ of the judgment matrix is calculated according to Equation (5) and is approximately equal to 4.004, where n indicates the number of primary indicators. Then the corresponding consistency test is completed according to Equations (5) and (6). Then the consistency indicator CI of judgment matrix A is calculated by Equations (5) and (6) is approximately equal to 0.001, and RI is approximately equal to 0.90. Moreover, the ratio CR [13] between CI and RI is calculated according to Equation (6), and finally, according to Equation (7), the value is approximately 0.002, which is less than 0.1; then, the consistency of the judgment matrix satisfies the requirements, indicating that the weight allocation of this primary indicator is more scientific and reliable. Finally, the importance of the secondary indicators to the primary indicators is set to be equal, and the results of the weight distribution of the primary and secondary indicators of the evaluation indicator system by the AHP method.

(5) $$\lambda_{\max}=\frac{1}{n}\sum_{i=1}^{n}\frac{{\left(AW\right)}_{i}}{w_{i}}$$

(6) $$CI=\frac{\lambda_{\max}-n}{n-1}$$

(7) $$CR=\frac{CI}{RI}$$

Finally, the importance of the secondary indicators to the primary indicators is set to be equal, and the weights of the primary and secondary level indicators of the evaluation indicator system are assigned to the results by the AHP method where n indicates the judgment matrix order, CI refers to the judgment matrix consistency indicator, RI is the average random consistency indicator value of the same order. RI is taken according to the judgment matrix order, and the values are shown in Table 3 below, and CR refers to the random consistency ratio.

### 3.2. Entropy Method

The entropy method is an evaluation method based on information entropy, which can be used as the basis for indicator assignment by calculating the information entropy size of each indicator to carry out the assignment of indicator weights and finally arrive at an objective and scientific evaluation result. Many studies have applied the entropy method to the objective assignment process of the comprehensive evaluation indicator system and then combined it with comprehensive evaluation models such as TOPSIS to conduct a comprehensive evaluation of the evaluation object [38,39]. Some other studies directly applied the entropy method for a comprehensive evaluation by multiplying the standardized indicators with their corresponding weight results and then summing the results to directly obtain the evaluation score corresponding to each evaluation object, thus completing the comprehensive evaluation [40].

(1) Considering that there are some differences between the attributes of different environmental performance evaluation indicators, the environmental performance evaluation indicators with negative attributes are first positivized according to Equation (8). Among them, $q_{ij}$ refers to the negative indicator, $t_{ij}$ refers to the positive indicator and the negative indicator which has been positivized.

(8) $$t_{ij}=\max(q_{ij})-q_{ij}$$

(2) In order to standardize the scale of the processed environmental performance evaluation indicators, the data were standardized in this study, as shown in Equation (9).

(9) $$x_{ij}=\frac{t_{ij}-\min(t_{ij})}{\max(t_{ij})-\min(t_{ij})}$$

(3) Based on the indicator data calculated in Equation (9), the comparative information entropy $e_{j}$ of each environmental performance evaluation indicator is calculated in Equation (10), as well as combined with Equation (11) to calculate its corresponding pair of information utility values $d_{j}$.

(10) $$e_{j}=\frac{1}{\ln n}\sum_{i=1}^{n}P_{ij}\ln(x_{ij})$$

(11) $$d_{j}=1-e_{j}$$

(4) Finally, the utility value obtained from Equation (12) is used as the basis for assigning weights to each indicator so that the jth environmental performance evaluation indicator is assigned an objective weight value $w_{j}$ corresponding to it.

(12) $$w_{j}=\frac{d_{j}}{\sum_{j=1}^{m}d_{j}}$$

### 3.3. Portfolio Empowerment

The integrated algorithm of Equation (13) is used in this paper to combine the results of the above calculations obtained by using the subjective and objective methods of weighting, respectively, according to Equations (14) and (15), where *ζ* and *η* are coefficients to be determined, which ultimately reflect the specific proportion of the subjective and objective weights in the combined weighting calculation.

(13) $$W=\xi W_{s}+\eta W_{o}$$

(14) $$\xi =\frac{n}{n-1}[\frac{2}{n}(P_{1}+2P_{2}+\cdots +nP_{n})-\frac{n+1}{n}]$$

(15) η=1−ξ

In Equation (13), W is the overall subjective and objective combination weight, $W_{s}$ is the subjective weight determined by the AHP method, and $W_{o}$ is the objective weight calculated by the entropy assignment method; in Equation (14), $P_{i}$ is the component of the subjective weight vector corresponding to the ascending order; n is the number of indicator terms.

Through the calculation of Equations (13)–(15), the proportion of the subjective and objective weights of the primary and secondary indicators in Table 4 is derived, and the specific results of the combined weights of the second-level indicators corresponding to this multiple urban environmental performance evaluation indicator system are finally obtained, as shown in Table 5.

### 3.4. Norms and Rules for Judging Indicators

In this paper, the primary indicators of the multi-city environmental performance evaluation system are selected downwards, and 24 effective secondary indicators are assigned a combination of weights to determine the influence weight of each indicator on the overall urban environmental performance, so the key issue in constructing this multi-city environmental performance evaluation system lies in the determination of the specific numerical merits of the above 24 secondary indicators. In this paper, concerning the existing national standards for urban requirements at all levels, industry norms, planning documents, and other policies and regulations, the specific criteria of the relevant documents for the environmental performance evaluation indicators of the twin cities of Chengdu and Chongqing are clarified, and their parameters are assessed and judged, and the specific evaluation criteria and rules are shown in Table 6.

## 4. Results and Discussion

This paper combines the above-mentioned criteria and rules to determine the 24 secondary indicators for the period 2011–2020 in Chengdu and applies the criteria of attainment and non-attainment, i.e., indicators that attain the criteria are assigned a score corresponding to their weights, while those that do not attain the criteria are not assigned any score. In particular, for some of the indicators in the evaluation system, as there is no unified international and domestic standard, this paper chooses to use the average value of this indicator between 2011 and 2020 as the standard instead to quantify and compare the environmental performance of the Chengdu-Chongqing twin cities as a whole and the environmental subsystems between 2011 and 2020.

**Figure 3:**
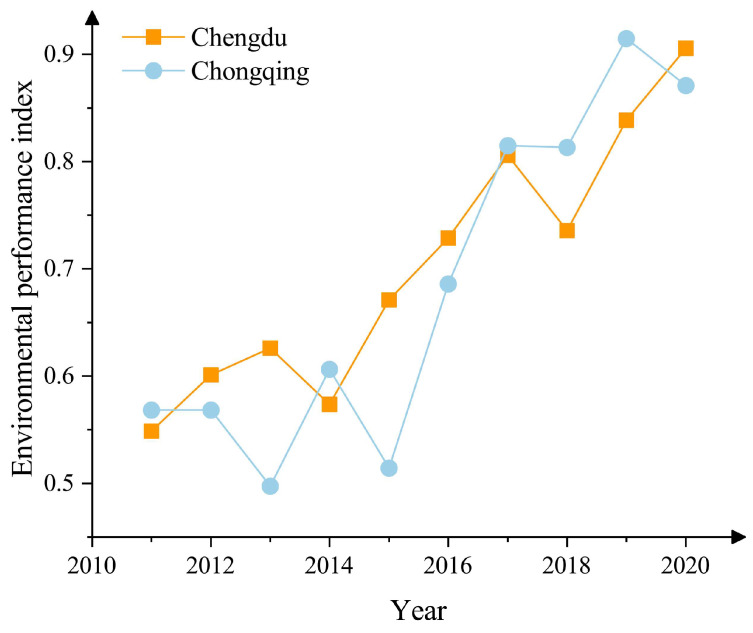
Overall development trend of environmental performance indicator.

The overall trend of change in the environmental performance indicator of the Chengdu-Chongqing region from 2011 to 2020, given in Figure 3, shows that: between 2011 and 2015, the environmental performance indicator of Chengdu City grew at a relatively slow pace, and although there was a decline in 2014, it did not fall below the environmental performance indicator of 2011, and still showed an overall trend of growth. In contrast, Chongqing’s environmental performance indicator had two large drops between 2011 and 2015, reflecting from the calculation results that the overall environmental level of Chongqing did not improve significantly during this period. Between 2016 and 2020, as the state made it clear in 2016, the Chengdu-Chongqing urban agglomeration should be established as a national urban agglomeration by 2020 [47]. Based on the high importance attached by the national macro strategy to the environment of the Chengdu-Chongqing region, which has led to the continuation of a steady upward trend in Chengdu and a more substantial improvement in the environmental level of Chongqing, the environmental performance of Chengdu and Chongqing has now basically tended to develop in a synergistic and spiraling trend.

**Figure 4:**
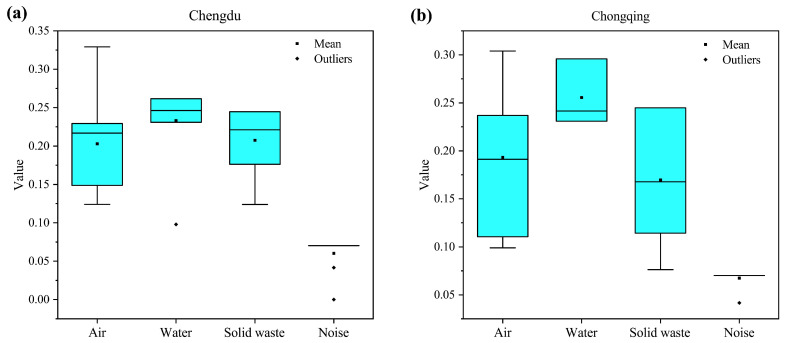
Boxplot of environmental performance indicator of each subsystem.

In order to facilitate subsequent trend analysis of each city subsystem individually, the outlier test was applied to each city environmental subsystem using the outlier quartile method, and the water environmental performance indicator for 2014 for Chengdu, the sound environmental performance indicator for 2016 and 2018, and the sound environmental performance indicator for 2013 for Chongqing were excluded from the test results. In addition, as most of the data for the sound environment subsystem are very stable, it is not meant to study the internal information of the data by plotting boxplots, so the analysis for the sound environment subsystem is chosen to be ignored here. The comparison of the average levels of each subsystem between Chengdu and Chongqing is shown in Figure 4. Among them, Figure 4a shows that the boxplot of Chengdu, and Figure 4b shows that the boxplot of Chongqing. Firstly, the average level of the environmental performance of the air environment subsystem and solid waste subsystem in Chengdu is lower but has a slight advantage over Chongqing, while Chongqing has a better average level of environmental performance in the water environment subsystem than Chengdu, and also has the highest average level of each environmental subsystem in the Chengdu-Chongqing twin cities. Secondly, the distribution of the data for each subsystem shows that the median of each environmental subsystem in Chengdu is greater than the mean and has a left-skewed distribution, indicating that the performance indices of each environmental subsystem in Chengdu over the past ten years are mostly above the average, while the mean of each environmental subsystem in Chongqing exceeds its median to varying degrees and has a right-skewed distribution. This means that the environmental performance of Chongqing’s environmental subsystems over the past ten years has been mostly below their average levels. Finally, a comparative analysis of the box heights of the environmental subsystem box plots of Chengdu and Chongqing shows that the data of Chongqing’s environmental subsystems fluctuate more than those of Chengdu, indicating that the overall stability of Chongqing’s subsystem environmental performance indices is poor, suggesting that Chongqing should make more efforts in urban environmental management in the future and steadily improve the level of urban ecological and environmental development.

**Figure 5:**
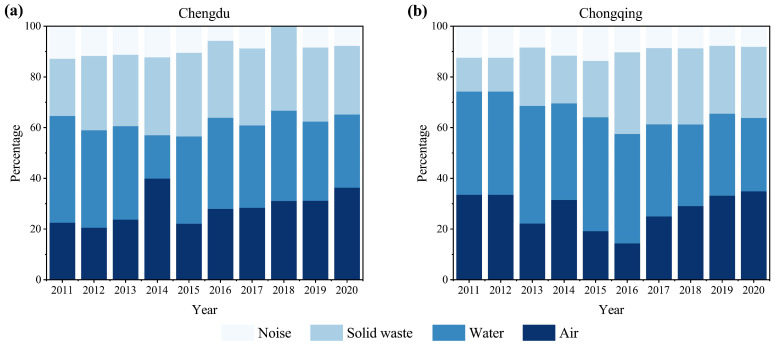
Percentage stacked histogram of the performance indicator of each environmental subsystem.

Figure 5 shows the percentage overlay histogram of environmental indices for Chengdu (Figure 5a) and Chongqing (Figure 5b), which reflects the trend of each environmental subsystem’s contribution to the city’s overall environmental performance from 2011 to 2020. Overall, the air and water environments contribute more to urban environmental performance than the other two subsystems, dioxygen, solid waste, and finally, noise, indicating that noise has a lower impact on the level of urban environmental performance. From the trends of the contribution of each subsystem to urban environmental performance in Chengdu and Chongqing, there are some similarities and differences in their fluctuation characteristics. The same change characteristics show that the contribution of water and noise environments to overall urban environmental performance is decreasing in both Chengdu and Chongqing, while the influence of solid waste on urban environmental performance is gradually increasing and stabilizing. This indicates that the impact of water and noise environments on urban environmental performance is gradually diminishing in Chengdu and Chongqing, and greater attention should be given to solid waste emissions in the future. In contrast, the contribution rate of the air environment in Chengdu tends to increase, while in Chongqing, it follows a pattern of “decreasing first, then increasing.” It is visible that although the fluctuation characteristics of the air environment contribution rate are different in Chengdu and Chongqing, it does not affect the objective fact that the air environment has an important impact on the overall urban environmental performance. In the future, the air environment will still be the key influencing factor for improving urban environmental performance levels. In addition, it is worth noting that since 2019, the contribution rate of each environmental subsystem to the overall urban environmental performance in Chengdu and Chongqing has tended to be stable and coordinated, indicating that the double-city collaborative development strategy implemented by the local government has begun to take effect and has strong potential for transformation to higher quality development.

**Figure 6:**
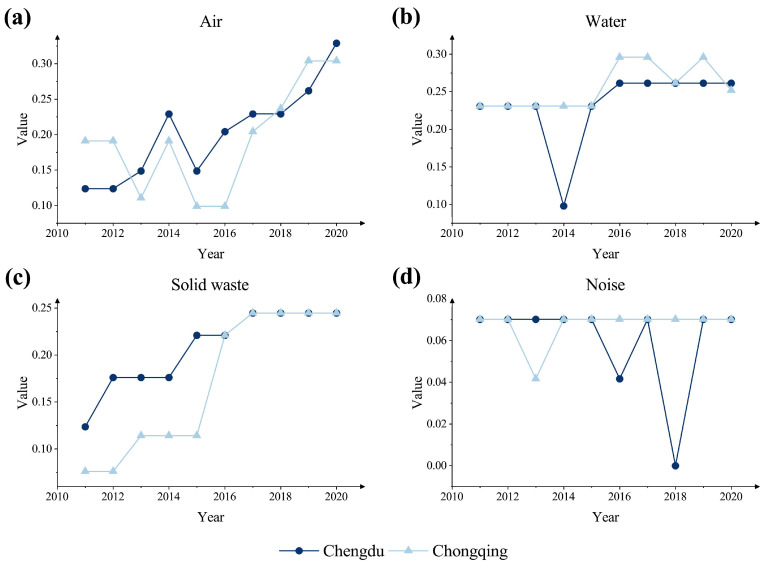
Trend of each environmental subsystem.

The trends of each subsystem of environmental performance evaluation in the Chengdu-Chongqing twin cities from 2011 to 2020 are shown in Figure 6. Among them, Figure 6a refers to the air, Figure 6b refers to the water, Figure 6c refers to solid waste, and Figure 6d refers to the noise. The results show that in terms of air environment, the air environment indicator of both cities maintains a coordinated upward trend, indicating that the linkage mechanism of both cities in air pollution prevention and control is becoming increasingly close. Specifically, the air quality in Chongqing City showed a significant decrease in 2013, mainly due to the increase of the large PM10 concentration from 90 μg/m^3^ in 2012 to 106 μg/m^3^ in 2013, which was far higher than the standard of 70 μg/m^3^ for PM10 [41], indicating that the city had relatively serious problems in air quality that year, which was also a major factor leading to the decrease of the air quality in Chongqing City. In order to further improve the urban air quality and strengthen the quality of the urban air environment, the State Council issued and implemented the Action Plan for Prevention and Control of Air Pollution in September 2013, and Chongqing City subsequently issued the Implementation Opinions of Chongqing Municipal People’s Government on the Implementation of the Action Plan for Prevention and Control of Air Pollution. Chongqing City has been making stricter and more effective management of its air environment by adjusting and optimizing the industrial and energy structure, upgrading the environmental access conditions for projects, strengthening pollution control, and strictly monitoring pollution emissions. At the same time, this series of initiatives has led to a significant improvement in Chongqing’s air environment since 2014. In addition, the Chongqing Ecological Environment Bureau issued the Chongqing Air Quality Automatic Monitoring Station Operation and Maintenance Supervision and Management Four-party Coordination Work Mechanism in 2016, which further strengthened the detection of air quality in Chongqing, improved the air quality supervision and maintenance mechanism, and achieved timely prevention and control of air pollution, so that since 2017, the air environmental quality has a significant upward trend.

In terms of the water environment, the water environment in Chengdu City experienced a sharp decline in 2014 but quickly recovered in 2015 and then stabilized in 2016–2020. This result occurred mainly since the total of the city’s water reuse rate and water saving rate did not meet the standard, indicating that the residential water use in Chengdu City was not ideal. Therefore, Chengdu City issued the Regulations on Water Source Protection for Drinking Water in 2014, which effectively protected the safety of residential water use, improved the efficiency of residential water supply in the city, and thus made the water environment quality in Chengdu City rebound in 2015. The trend of changes in Chongqing City in 2014 was stable from 2011 to 2015 and generally rose from 2016 to 2020.

In terms of solid waste emissions, Chengdu and Chongqing have roughly the same trend of change, both showing step-up development, especially from 2016 to 2020, when the solid waste indices of the two cities tend to be the same. Specifically, during this period, the solid waste indicator of Chongqing City increased more, indicating that Chongqing City is more effective in improving its municipal solid waste emission management compared to Chengdu City. Concerning the noise environment, ignoring the effect of outliers on the overall trend, Chengdu and Chongqing have remained stable at a relatively good level over the past 10 years, and have met the current urban noise-related limit standards implemented by the state in the vast majority of years.

Based on the specific analysis of the components of the city’s multifaceted environmental performance evaluation system above, the environmental performance of the Chengdu-Chongqing region is predicted in four separate components. The first among them is the air environment. By comparing the growth rate of the air environment performance of Chengdu and Chongqing from 2011 to 2020, it is found that Chongqing’s air environment indicator gradually stabilized from 2019, while Chengdu still showed a continuous growth trend and the slope from 2019 to 2020 increased significantly compared to 2018 to 2019. It is expected that Chengdu will gradually expand its leading-edge compared to Chongqing; the second is the water environment. As shown in Figure 6b, the water environment performance indicator of Chengdu was in a stable state from 2016 to 2020 and is not expected to change significantly soon; while for Chongqing, the city’s water environment performance may bottom out in the future as the national macro-strategy will pay more and more attention to urban environmental management to promote high-quality economic development. Then there is solid waste. According to the above analysis of the solid waste environmental performance indices of Chongqing and Chengdu, there is a synergistic stepwise increase in the effectiveness of solid waste management in both cities, but the results reflected in Figure 6c show that there is a certain lagging effect in the change of Chongqing’s solid waste environmental performance compared to that of Chengdu, indicating that Chongqing’s solid waste environmental performance has taken longer to respond to policies than that of Chengdu. Therefore, it is expected that the environmental performance of solid waste in Chongqing and Chengdu will continue to grow in the future and that Chengdu will be more responsive to policies. Finally, in terms of the four components is the sound environment. This paper, combined with the historical data, concludes that without drastic changes in the surrounding political and economic ecological environment, there will be no significant fluctuations in the short term, and the sound environment performance evaluation indicator will remain at around 0.07. On the whole, through the deepening cooperation between the two cities, the ecological environment of Chengdu and Chongqing will show alternate growth and green synergy in the future with high-quality development.

Aside from this, this study also took Chengdu and Chongqing as examples to study the impact of COVID-19 on urban environmental performance, providing new references and evidence for related fields. In terms of the overall environmental performance level of the city, the effect of COVID-19 on Chengdu and Chongqing may be different. Specifically, COVID-19 may have a positive effect on the environmental performance of Chengdu, while it may harm the environmental performance of Chongqing. To further explore the reasons for the difference in the direction of the overall indicator fluctuation, this study further analyzed the indicator of each subsystem. From the change of the indicator of each subsystem, the air environment indicator is the most affected by the impact. The main reasons are: first, the restriction of travel and consumption activities of urban residents during the epidemic period led to a decrease in the total amount of air pollutants emitted from urban transportation and other aspects; second, during the epidemic period, large-scale shutdowns or production restrictions of industrial production enterprises reduced the total amount of industrial pollutants emitted to the atmosphere. As for the solid waste and noise environment indicator, although the total amount of solid waste emission decreased due to the large-scale shutdown of industries during the epidemic period, the urban noise was relatively low due to the restrictions on the residents’ travel. Since the solid waste and noise environment indicator of Chengdu and Chongqing had reached the standard before 2019, the epidemic did not have a great impact on the indicator. In addition, the water environment is the main reason for the decline of Chongqing’s environmental performance level, and the main reason for the negative impact on the water environment indicator is the unqualified indicator of urban industrial water use. The reason for this result may be that the supervision of industrial water use was relaxed during the epidemic period. Therefore, Chongqing should strengthen the supervision of industrial water use in the future and improve the water use efficiency of industrial enterprises.

## 5. Conclusions

This research takes the twin cities of Chengdu and Chongqing as the research object and uses urban environmental evaluation indicators to establish a multifaceted urban environmental performance evaluation system for the Chengdu and Chongqing region in four dimensions: air environment, water environment, solid waste, and noise environment and quantitatively reflects the urban environmental performance with an environmental performance indicator to evaluate the environmental performance of the Chengdu and Chongqing region from 2011 to 2020. The results of the final analysis and comprehensive evaluation objectively reflect the efforts made by the twin cities of Chengdu and Chongqing in environmental management and the remarkable results achieved from 2011 to 2020. Moreover, this paper explores the impact of COVID-19 on urban environmental performance. In general, from the development of the overall Chengdu-Chongqing multiple environmental performance system, different environmental performance subsystems show some differences in values, which mainly reflect that urban water environmental quality is the best, air environmental quality and solid waste management are the second best, urban noise environmental quality remains relatively stable, and the overall level of the Chengdu-Chongqing multiple environmental systems is on the rise. In the cross-sectional comparison between Chengdu and Chongqing, Chengdu is better than Chongqing in terms of air environment quality and solid waste management, while Chongqing is better than Chengdu in terms of urban water environment and noise environment quality. In addition, for the impact of COVID-19 on urban environmental performance, this paper compares the environmental performance evaluation results of Chengdu and Chongqing cities and finds that the positive impact of the epidemic on the air environment causes the environmental performance level of Chengdu to increase in 2020, while the water environment evaluation result of Chongqing city decreases slightly due to the possible relaxation of the regulation of industrial enterprises’ water use during this period, which led to a decline in its overall urban environmental performance level. Therefore, in the future environmental management of Chengdu and Chongqing, on the one hand, cities can refer to the existing evaluation results, be more proactive to make up for their shortcomings, pay more attention to the aspects that have a strong contribution to the urban environmental performance level, especially strengthen the supervision of residential and industrial water use, and improve the overall water use efficiency of the city. On the other hand, by deepening the joint action of the Chengdu-Chongqing region, the two places can formulate joint environmental governance policies, strive to complement each other’s advantages, continuously enhance the coherence of environmental development in Chengdu and Chongqing, focus on the development of urban agglomerations, and further improve the ecological environment of Chengdu-Chongqing region and build a green and high-quality development of Chengdu-Chongqing economic circle.

## Figures and Tables

**Table 1:** The indicator system of environmental performance evaluation.

Target	Primary Indicators	Secondary Indicators	Unit	Type
Environment Performances	Air (A)	PM10 Annual average concentration (A1)	μg/m^3^	-
		${\mathrm{NO}}_{2}$ Annual average concentration (A2)	μg/m^3^	-
		${\mathrm{SO}}_{2}$ Annual average concentration (A3)	μg/m^3^	-
		Average annual temperature (A4)	°C	-
		Number of days with the air quality of Grade 2 or above (A5)	%	+
		Annual share of good air quality days (A6)	Number of days	+
		The treatment capacity of exhaust gas treatment equipment (A7)	Million m^3^/h	+
	Water (B)	The urban water reuse rate (B1)	%	+
		Water conservation rate for urban water supply (B2)	%	+
		The urban industrial water reuse rate (B3)	%	+
		Water conservation rate in urban industry (B4)	%	+
		Combined water consumption per capita (B5)	Liters per person. Day	-
		Water consumption per GDP (B6)	Tons/million RMB	-
		Unit urban wastewater discharge intensity (B7)	Tons/million RMB	-
		Unit urban industrial effluent discharge intensity (B8)	Tons/million RMB	-
	Solid waste (C)	Industrial solid waste generation (C1)	Million tons	-
		Comprehensive utilization of industrial solid waste (C2)	Million tons	+
		The integrated utilization rate of industrial solid waste (C3)	%	+
		Total domestic waste removal (C4)	Million tons	+
		Harmless disposal rate of domestic waste (C5)	%	+
		Household waste removal per capita (C6)	Kg/person. Day	+
		Household waste disposal capacity (C7)	Tons/day	+
	Noise (D)	Urban road traffic noise (D1)	dB(A)	-
		Urban ambient noise (D2)	dB(A)	-

Note: The indicators in the table are the standard for evaluating the urban environmental performance of Chengdu and Chongqing cities from 2011 to 2020. Among them, A refers to the urban air environment situation, B refers to the urban water environment situation, C refers to the urban solid waste situation, and D refers to the urban noise situation. Furthermore, the type “+” refers to the positive indicator, and the type “-” refers to the negative indicator.

**Table 2:** Expert judgment matrix after taking mean values.

Judgment Matrix	Air	Water	Solid Waste	Noise
Air	1	1.072	2.048	1.966
Water	0.933	1	2.107	2.024
Solid waste	0.488	0.475	1	1.123
Noise	0.509	0.494	0.891	1

**Table 3:** The value of RI.

*n*	1	2	3	4	5	…	9	10
*RI*	0.00	0.00	0.58	0.90	1.12	…	1.45	1.49

**Table 4:** Weighting factors for the combination of indicators at each level.

	*ζ*	*η*
Primary indicators	0.233	0.767
Secondary indicators	0.193	0.807

**Table 5:** Subjective and objective indicators of the multi-city environmental performance evaluation system, as well as the results of the combination of weighting.

Primary Indicators	Secondary Indicators	Subjective Weights	Objective Weights	Portfolio Weights
Air (A)	PM10 Annual average concentration (A1)	4.84%	2.84%	3.22%
	${\mathrm{NO}}_{2}$ Annual average concentration (A2)	4.84%	7.05%	6.63%
	${\mathrm{SO}}_{2}$ Annual average concentration (A3)	4.84%	4.15%	4.29%
	Average annual temperature (A4)	4.84%	1.92%	2.48%
	Number of days with air quality of Grade 2 or above (A5)	4.84%	2.25%	2.75%
	Annual share of good air quality days (A6)	4.84%	2.25%	2.75%
	The treatment capacity of exhaust gas treatment equipment (A7)	4.84%	11.71%	10.39%
Water (B)	The urban water reuse rate (B1)	4.15%	6.95%	6.41%
	Water conservation rate for urban water supply (B2)	4.15%	7.32%	6.71%
	The urban industrial water reuse rate (B3)	4.15%	4.38%	4.33%
	Water conservation rate in urban industry (B4)	4.15%	2.37%	2.71%
	Combined water consumption per capita (B5)	4.15%	3.46%	3.59%
	Water consumption per GDP (B6)	4.15%	2.27%	2.63%
	Unit urban wastewater discharge intensity (B7)	4.15%	5.76%	5.45%
	Unit urban industrial effluent discharge intensity (B8)	4.15%	2.77%	3.04%
Solid waste (C)	Industrial solid waste generation (C1)	2.40%	4.92%	4.43%
	Comprehensive utilization of industrial solid waste (C2)	2.40%	4.56%	4.14%
	The integrated utilization rate of industrial solid waste (C3)	2.40%	2.34%	2.36%
	Total domestic waste removal (C4)	2.40%	5.21%	4.67%
	Harmless disposal rate of domestic waste (C5)	2.40%	1.87%	1.97%
	Household waste removal per capita (C6)	2.40%	3.05%	2.92%
	Household waste disposal capacity (C7)	2.40%	5.83%	5.17%
Noise (D)	Urban road traffic noise (D1)	8.05%	1.59%	2.84%
	Urban Ambient Noise (D2)	8.05%	3.18%	4.12%

Note: The indicators in the table are the standard for evaluating the urban environmental performance of Chengdu and Chongqing cities from 2011 to 2020. Among them, A refers to the urban air environment situation, B refers to the urban water environment situation, C refers to the urban solid waste situation, and D refers to the urban noise situation.

**Table 6:** Evaluation criteria and judgment rules for secondary indicators.

Secondary Indicator Parameters	Unit	Evaluation Criteria and Judgement Rules
PM10 Annual average concentration	$\mu g/m^{3}$	Annual average concentration 70 $\mu g/m^{3}$ [41]
${\mathrm{NO}}_{2}$ Annual average concentration	$\mu g/m^{3}$	Annual average concentration 40 $\mu g/m^{3}$ [41]
${\mathrm{SO}}_{2}$ Annual average concentration	$\mu g/m^{3}$	Annual average concentration 60 $\mu g/m^{3}\;$ [41]
Average annual temperature	℃	Average 2011–2020
Number of days with air quality of Grade 2 or above	%	Greater than 213 days [42]
Annual share of good air quality days	Number of days	Greater than 58.6% [42]
The treatment capacity of exhaust gas treatment equipment	Million $m^{3}$/h	Average 2011–2020
The urban water reuse rate	%	Combined water savings of >25% with urban water supply
Water conservation rate for urban water supply	%	Combined water savings of >25% with urban water supply
The urban industrial water reuse rate	%	Combined with urban industrial water savings of >50%
Water conservation rate in urban industry	%	Combined with urban industrial reuse rate greater than 50%
Combined water consumption per capita	Liters per person. Day	Less than 134.8 Liters per person. Day [43]
Water consumption per GDP	Tons/million RMB	Less than 57.6 Tons/million RMB [43]
Unit urban wastewater discharge intensity	Tons/million RMB	Average 2011–2020
Unit urban industrial effluent discharge intensity	Tons/million RMB	Average 2011–2020
Industrial solid waste generation	Million tons	Average 2011–2020
Comprehensive utilization of industrial solid waste	Million tons	Average 2011–2020
The integrated utilization rate of industrial solid waste	%	Greater than 80% [44]
Total domestic waste removal	Million tons	Average 2011–2020
Harmless disposal rate of domestic waste	%	100%
Household waste removal per capita	Kg/person. Day	Less than 1.12 kg/person. Day
Household waste disposal capacity	Tons/day	Greater than 0.55 million tons per day [45]
Urban road traffic noise	dB(A)	Less than 70 dB(A) [46]
Urban Ambient Noise	dB(A)	Less than 55 dB(A) [46]

Note: Indicator benchmarks are generally implemented regarding national standards, national and provincial planning documents, city grading standards, and other strict standards; some indicators are two combined indicators, individual indicators currently have no industry standards, so they are specifically stated in the table and replaced with average values.

## Data Availability

Not applicable.

## References

[1] Li W., Mauerhofer V. (2016). Behavioral patterns of environmental performance evaluation programs. J. Environ. Manag..

[2] Griggs D.T., Smith M.S., Rockström J., Öhman M.C., Gaffney O., Glaser G., Kanie N., Noble I., Steffen W., Shyamsundar P. (2014). An integrated framework for sustainable development goals. Ecol. Soc..

[3] Hák T., Janoušková S., Moldan B. (2016). Sustainable Development Goals: A need for relevant indicators. Ecol. Indic..

[4] Wang C.C., Geng L.N., Rodriguez-Casallas J.D. (2021). How and when higher climate change risk perception promotes less cli-mate change inaction. J. Clean. Prod..

[5] Jiang Z., Wang Z., Lan X. (2021). How environmental regulations affect corporate innovation? The coupling mechanism of mandatory rules and voluntary management. Technol. Soc..

[6] Liang Z., Zhang M., Mao Q., Yu B., Ma B. (2018). Improvement of Eco-Efficiency in China: A Comparison of Mandatory and Hybrid Environmental Policy Instruments. Int. J. Environ. Res. Public Health.

[7] Meng F.X., Guo J.L., Guo Z.Q., Lee J.C.K., Liu G.Y., Wang N. (2021). Urban ecological transition: The practice of ecological civi-lization construction in China. Sci. Total Environ..

[8] Yu C.-H., Wu X., Zhang D., Chen S., Zhao J. (2021). Demand for green finance: Resolving financing constraints on green innovation in China. Energy Policy.

[9] Zhang Z. (2017). A method of indicator-indicator coupling chain for two-step measurement of the threshold value and green de-gree of ecological civilization. China Popul. Resour. Environ..

[10] Zhang Q.-F., Tang X., Xiao Y., Xiang X., Huang H. (2023). Coordination of industrial structure and eco-efficiency in ecologically fragile areas: A case study of the Loess Plateau, China. J. Environ. Manag..

[11] Liang L.W., Wang Z.B., Li J.X. (2019). The effect of urbanization on environmental pollution in rapidly developing urban ag-glomerations. J. Clean. Prod..

[12] Feng T., Du H., Lin Z., Zuo J. (2020). Spatial spillover effects of environmental regulations on air pollution: Evidence from urban agglomerations in China. J. Environ. Manag..

[13] Chen T.T., Peng L., Wang Q. (2022). Response and multiscenario simulation of trade-offs/synergies among ecosystem services to the Grain to Green Program: A case study of the Chengdu-Chongqing urban agglomeration, China. Environ. Sci. Pollut. Res..

[14] Wei Q., Xu X., Yang C., Yang L. (2021). Research on the Coupling and Coordination Measurement of Technological Innovation and High-quality Economic Development in Chengdu-Chongqing Double-city Economic Circle. Sci. Technol. Prog. Policy.

[15] Ding R., Xu B., Zhang H. (2021). Can Urban Agglomeration Drive Regional Economic Growth?Empirical Analysis Based on Seven State-level Urban Agglomerations. Econ. Geogr..

[16] Wu M., Wu J., Zang C. (2020). A comprehensive evaluation of the eco-carrying capacity and green economy in the Guangdong-Hong Kong-Macao Greater Bay Area, China. J. Clean. Prod..

[17] Zhang M., Liu Y.M., Wu J., Wang T.T. (2018). Indicator system of urban resource and environment carrying capacity based on ecological civilization. Environ. Impact Assess. Rev..

[18] Mori K., Christodoulou A. (2012). Review of sustainability indices and indicators: Towards a new City Sustainability Index (CSI). Environ. Impact Assess. Rev..

[19] Wang S., Gao S., Li S., Feng K. (2019). Strategizing the relation between urbanization and air pollution: Empirical evidence from global countries. J. Clean. Prod..

[20] Xiao Y., Li Y., Tang X., Huang H., Wang R. (2022). Assessing spatial–temporal evolution and key factors of urban livability in arid zone: The case study of the Loess Plateau, China. Ecol. Indic..

[21] Xiao Y., Wang R., Wang F., Huang H., Wang J. (2022). Investigation on spatial and temporal variation of coupling coordination between socioeconomic and ecological environment: A case study of the Loess Plateau, China. Ecol. Indic..

[22] Liu Y., Eckert C.M., Earl C. (2020). A review of fuzzy AHP methods for decision-making with subjective judgements. Expert Syst. Appl..

[23] Lin R.-J. (2013). Using fuzzy DEMATEL to evaluate the green supply chain management practices. J. Clean. Prod..

[24] Yi X., Jue W., Huan H. (2021). Does economic development bring more livability? Evidence from Jiangsu Province, China. J. Clean. Prod..

[25] Xu C., Ke Y., Li Y., Chu H., Wu Y. (2020). Data-driven configuration optimization of an off-grid wind/PV/hydrogen system based on modified NSGA-II and CRITIC-TOPSIS. Energy Convers. Manag..

[26] Wu Y., Liao M., Hu M., Lin J., Zhou J., Zhang B., Xu C. (2020). A decision framework of low-speed wind farm projects in hilly areas based on DEMATEL-entropy-TODIM method from the sustainability perspective: A case in China. Energy.

[27] Liu T., Deng Y., Chan F. (2017). Evidential Supplier Selection Based on DEMATEL and Game Theory. Int. J. Fuzzy Syst..

[28] Hu X., Ma C., Huang P., Guo X. (2021). Ecological vulnerability assessment based on AHP-PSR method and analysis of its single parameter sensitivity and spatial autocorrelation for ecological protection—A case of Weifang City, China. Ecol. Indic..

[29] Levrel H., Kerbiriou C., Couvet D., Weber J. (2009). OECD pressure-state-response indicators for managing biodiversity: A re-alistic perspective for a French biosphere reserve. Biodivers. Conserv..

[30] Chen L., Huang H., Han D., Wang X., Xiao Y., Yang H., Du J. (2023). Investigation on the spatial and temporal patterns of cou-pling sustainable development posture and economic development in World Natural Heritage Sites: A case study of Jiu-zhaigou, China. Ecol. Indic..

[31] Zheng S., Cao C.-X., Singh R.P. (2014). Comparison of ground based indices (API and AQI) with satellite based aerosol products. Sci. Total Environ..

[32] Li C., Zwiers F., Zhang X.B., Li G.L., Sun Y., Wehner M. (2021). Changes in Annual Extremes of Daily Temperature and Precipi-tation in CMIP6 Models. J. Clim..

[33] Rezaei J. (2015). Best-worst multi-criteria decision-making method. Omega-Int. J. Manag. Sci..

[34] Pourghasemi H.R., Pradhan B., Gokceoglu C. (2012). Application of fuzzy logic and analytical hierarchy process (AHP) to land-slide susceptibility mapping at Haraz watershed, Iran. Nat. Hazards.

[35] Luthra S., Govindan K., Kannan D., Mangla S.K., Garg C.P. (2016). An integrated framework for sustainable supplier selection and evaluation in supply chains. J. Clean. Prod..

[36] Taylan O., Bafail A.O., Abdulaal R.M., Kabli M.R. (2014). Construction projects selection and risk assessment by fuzzy AHP and fuzzy TOPSIS methodologies. Appl. Soft Comput..

[37] Konstantinos I., Georgios T., Garyfalos A. (2019). A Decision Support System methodology for selecting wind farm installation locations using AHP and TOPSIS: Case study in Eastern Macedonia and Thrace region, Greece. Energy Policy.

[38] Li Z., Luo Z.J., Wang Y., Fan G.Y., Zhang J.M. (2022). Suitability evaluation system for the shallow geothermal energy imple-mentation in region by Entropy Weight Method and TOPSIS method. Renew. Energy.

[39] Chen P. (2020). Effects of the entropy weight on TOPSIS. Expert Syst. Appl..

[40] Huang H., Wang F., Xiao Y., Kuang J. (2021). Towards the Coupling Coordination Relationship between Economic Growth Quality and Environmental Regulation: An Empirical Case Study of China. Discret. Dyn. Nat. Soc..

[41] (2018). Ambient Air Quality Standards.

[42] Chengdu Development and Reform Commission (2017). The Thirteenth Five-Year Plan for the Construction of Ecological Civilization in Chengdu.

[43] (2007). Integrated Urban Water Consumption Standards.

[44] (2014). National Sanitary City Standards.

[45] National Development and Reform Commission (2016). The 13th Five-Year Plan for the Construction of Harmless Urban Domestic Waste Treatment Facilities in China.

[46] (2017). Environmental Noise Limits for the Sound Environment Functional Area.

[47] National Development and Reform Commission (2016). Chengdu-Chongqing Urban Agglomeration Development Plan.

